# Petroleum Depletion Property and Microbial Community Shift After Bioremediation Using *Bacillus halotolerans* T-04 and *Bacillus cereus* 1-1

**DOI:** 10.3389/fmicb.2020.00353

**Published:** 2020-03-05

**Authors:** Zhenshan Deng, Yingying Jiang, Kaikai Chen, Fei Gao, Xiaodong Liu

**Affiliations:** College of Life Sciences, Yan’an University, Yan’an, China

**Keywords:** petroleum contamination, bioremediation simulation tests, microbial community, high-throughput sequencing, petroleum biodegradation

## Abstract

Bioremediation of crude oil contaminated environments is an economical, low-maintenance, environmentally friendly technology and has attracted increasing attention in recent years. In the present study, two efficient crude oil degrading bacteria strains were isolated from soils contaminated with crude oil. Phylogenetic analysis suggested they belonged to genus *Bacillus*, and were designated as *Bacillus cereus* T-04 and *Bacillus halotolerans* 1-1. The crude oil depletion of each strain under different conditions was tested. The optimum conditions for both strains’ oil degradation was pH 7, 15–20 g/L NaCl concentration, and 5–15 g/L original oil concentration. The crude oil depletion rate could reach to 60–80% after 20 days of treatment. The crude oil bioremediation simulation tests revealed that the bioremediation promoted the depletion of crude oil to a large extent. The inoculum group with inorganic medium showed the highest crude oil depletion (97.5%) while the crude oil depletion of control group was only 26.6% after 180 days of treatment. High-throughput sequencing was used to monitor the changes of microbial community using different treatments. In all groups, Actinobacteria, Proteobacteria, Firmicutes and Bacteroidetes were the dominant phyla. After contaminated with crude oil, the relative abundance of phylum Actinobacteria was dramatically increased and occupied 81.8%. Meanwhile although strains of *Bacillus* were added in the bioaugmentation groups, the relative abundance of genus *Bacillus* was not the most abundant genus at the end of simulation tests. The crude oil contamination dramatically decreased the soil microbial diversity and bioremediation could not recover the microbial community in the short term.

## Introduction

As an essential source of energy and chemical raw materials, crude oil currently plays an important role in every aspect of the social life. However, the unexpected release of crude oil to the environment has caused serious environmental pollution. The high incidence and long-term environmental impact of crude oil make petroleum contamination a threat to living creatures, including humans and the environmental microbial community balance (Li et al., 2015). Due to the serious damage to the environment, several techniques have been introduced to deplete the crude oil contamination, including physical and chemical methods (Riser-Roberts, 1998). However, there are many limitations of the physical and chemical techniques – they are expensive, usually only partially effective, and often environmentally unfriendly (Riser-Roberts, 1998). Thus, bioremediation has been introduced and received a great deal of attention as an economical, environmentally friendly technique that is suitable for many situations (Harayama et al., 2004). Since bioremediation was introduced to deplete crude oil contamination, many microorganisms have been confirmed to degrade crude oil (Adams et al., 2015; Vinothini et al., 2015; Zhang et al., 2016; Ke et al., 2019).

Although crude oil contamination can enrich the native crude oil degraders, the process will last a long period of time and come with large ecological costs. Thus, bioremediation – including biostimulation and bioaugmentation – can accelerate the rate of crude oil depletion and is essential for crude oil removal (Adams et al., 2015). The biodegradation of petroleum-contaminated soil was compared by three methods (natural attenuation, biostimulation, and bioaugmentation), and the results showed that bioaugmentation possessed the highest degradation in the light and heavy fractions of petroleum (Bento et al., 2005). Another research also showed the higher bioremediation efficiency of bioaugmentation and phytoremediation than natural attenuation (Cai et al., 2016). In the bioaugmentation progress, efficient crude oil degrading bacteria are the key point. Many crude oil degrading bacteria had been isolated and the crude oil degrading properties under different conditions were also tested (Morikawa et al., 1993; Dave et al., 2014; Deng et al., 2014; Vinothini et al., 2015; Zhang et al., 2016). A pilot-scale study of bioremediation demonstrated that the crude oil degradation can reach up to 76% and the treated soil can support the germination and growth of crop plants (Mukherjee and Bordoloi, 2011).

The soil microorganism community is an important soil health indicator. Crude oil contamination can change the microbial community and have negative effects on other living creatures (Harayama et al., 2004; Gao et al., 2015; Jiao et al., 2016). The microorganisms used in the bioremediation process can also have effects on the native microbial community (Jung et al., 2016; Wu et al., 2017; Roy et al., 2018). Taking advantage of high-throughput sequencing technology, it is now convenient and economical for researchers to investigate microbial communities in diverse environments. Moreover, the 16S rDNA variable regions are usually used in high-throughput sequencing to investigate the microbial community structure because they can reflect the overall microbial community compared with traditional methods such as bacteria isolation and denaturing gradient gel electrophoresis (DEEG).

In the present study, two efficient crude oil degraders (i.e., strains *Bacillus cereus* T-04 and *Bacillus halotolerans* 1-1) were isolated from soil contaminated by crude oil. The growth and crude-oil-degradation properties were investigated, and a simulated bioremediation of crude-oil-contaminated soil was also conducted under laboratory conditions. The microbial community shift in the simulated bioremediation tests was investigated using Illumina high-throughput sequencing. The main objectives of this study were to: (1) investigate the crude oil degradation properties of the two isolated degraders; and (2) explore the microbial community shift of the crude-oil-contaminated soil under exogenous bacterial bioremediation. In this paper, we provided two efficient crude oil degrading *Bacillus* strains and a preliminary understanding of the microbial community shift by different bioaugmentation methods.

## Materials and Methods

### Materials

The crude-oil-contaminated soil was collected from an oil field located in Yanchang County, north of Shannxi province, and was used for crude oil degrader isolation. The physicochemical characteristics of the contaminated soil are listed in Table 1. Crude oil was provided by a refinery corporation from Ansai, Shannxi province. The crude oil mainly consisted of n-alkanes. The soil used for the laboratory simulation crude oil bioremediation test was collected from the hill at a depth of 30 cm behind Yan’an University, which was not contaminated with crude oil. The collected soil was air dried and filtered using a 2 mm sieve and then stored at 4°C in the dark. The soil used for simulated crude oil degradation tests was designated as clean soil. All chemicals used for bacterial culture and crude oil measurement were of analytical grade or better, and were purchased from Sinopharm Chemical Reagent Co., Ltd., China.

**TABLE 1:** Physicochemical characteristics of the contaminated soils^#^ used for isolation of crude-oil-degrading strains.

**Altitude**	**Latitude and longitude**	**Total N Content (%)**	**Organic C Content (%)**	**C/N**	**Total K Content (mg/kg)**	**Total P Content (mg/kg)**	**Soil pH**
1371.9 m	37°04′45″N, 109°05′27″E	0.015	16.88	10.4	61.4	1.37	8.14

*^#^Total nitrogen: Kjeldahl method; Soil organic carbon: K_2_CR_2_O_7_ volumetric analysis; Total P: acid-soluble molybdenum antimony colorimetry resistance; Total potassium: extraction – flame photometer method; Soil pH value: potentiometry.*

The crude-oil-degrading bacteria isolation medium contained 10 mL of crude oil, 6.0 g of KNO_3_, 5.0 g of K_2_HPO_4_, 5.0 g of KH_2_PO_4_, and 5 mL trace element solution. The trace element solution consisted of 5 g of FeSO_4_⋅7H_2_O, 5 g of MnSO_4_⋅H_2_O, and 5 g of CaCl_2_ per liter. The Luria–Bertani (LB) medium contained 10.0 g of peptone, 5.0 g of yeast extract, and 10.0 g of NaCl per liter. The mineral salt (MS) medium contained 10.0 g of NaCl, 1.0 g of K_2_HPO_4_, 0.5 g of KH_2_PO_4_, 0.5 g of NH_4_Cl, 0.5 g of MgSO_4_⋅7H_2_O, 0.02 g of CaCl_2_, and 0.1 g of KCl per liter. The organic nutrient (MO) medium contained 10.0 g of peptone and 3.0 g of lecithin per liter. The mixed nutrient (MX) medium contained 8.0 g of NaCl, 1.0 g of K_2_HPO_4_, 0.5 g of KH_2_PO_4_, 0.5 g of NH_4_Cl, 0.5 g of MgSO_4_⋅7H_2_O, 0.02 g of CaCl_2_, 0.1 g of KCl, 5.0 g of peptone, and 1.0 g of lecithin per liter. For solid media, agar was added into the solution at the final concentration of 15 g/L. The pH was adjusted to neutral using 1.0 M NaOH before autoclaving at 121°C for 20 min. For pH adjustment, 1.0 M NaOH or 1.0 M HCl were used to adjust the original pH of the media.

### Methods

#### Isolation of Crude-Oil-Degrading Bacteria

Crude-oil-degrading strains were isolated using the dilution plating method. The isolation medium containing crude oil as the sole carbon source was used for screening the crude-oil-degrading bacteria.

#### Identification of the Crude-Oil-Degrading Isolates

After their purification, the isolates were cultured on LB medium for 3 days. Then, colony PCR was used to identify the bacterial strain. Briefly, about 2 μL of bacteria were picked into a 50 μL 2 × T5 Super PCR Mix (Colony) system (TSINGKE Biological Technology, Beijing, China) and then amplified and sequenced using primers 27F (5′-AGAGTTTGATCCTGGCTCAG-3′) and 1492R (5′-GGTTACCTTGTTA CGACTT-3′). The 16S rRNA gene sequences were submitted to GenBank and blasted using EzBioCloud (Yoon et al., 2017). The 16S rRNA gene sequences were analyzed using MEGA version 5.1 and the phylogenetic tree was constructed based on neighbor-joining (Tamura et al., 2011).

#### Crude Oil Degradation Properties Under Different Conditions

The crude oil degradation efficiencies of the isolates were tested under different original pH values (3.0, 4.0, 5.0, 6.0, 7.0, 8.0, and 9.0 with a crude oil concentration of 5 g/L and salt concentration of 20.0 g/L), original crude oil concentrations (5, 10, 15, 20, 30, 40, and 50 g/L, with pH 7.0 and salt concentration of 20.0 g/L), and original salt concentrations (0, 10, 15, 20, 30, 40, 50, and 60 g/L, with pH 7.0 and crude oil concentration of 5 g/L). All tests were conducted using 100 mL mineral salt medium with 1 mL of inoculum in a 250 mL flask at 150 rpm and 30°C. The crude oil degradation rate was detected after 20 days. The crude oil was extracted by CCl_4_ and dried using Na_2_SO_4_, and the concentration was measured by an infrared spectrometer oil content analyzer (OIL 480, Beijing ChinaInvent Instrument Co., Ltd., China).

#### Simulation Tests of Crude Oil Degradation

For simulation tests of the crude oil degradation, sterile plastic boxes with 2 kg of clean soil were used. Twenty grams of crude oil was added into the clean soil and mixed uniformly. The inoculum was prepared using strains T-04 and 1-1 cultured in LB medium to the exponential growth phase. The bacteria were collected for the following usage. The simulation tests could be divided into five groups: (1) contaminated soil plus 200 mL sterilized water (designated as CW); (2) bacterial inoculum with 200 mL MS medium (designated as BAI); (3) bacterial inoculum with 200 mL MO medium (designated as BAO); (4) bacterial inoculum with 200 mL MX medium (designated as BAM); and (5) pure bacteria with 200 mL sterile water (designated as BAB). The inoculum was added to the crude-oil-contaminated soil and mixed uniformly. The water content was maintained at about 20% for each box. Samples were collected at 20, 60, 90, 120, and 180 days. The crude oil contents were tested as described in section Crude Oil Degradation Properties Under Different Conditions.

#### Bacterial Community Shift in the Crude Oil Bioremediation

Soil samples (0.5 g) for each group of crude oil bioremediation simulation tests were collected at 180 days and used for total DNA isolation. The total DNA of each sample was extracted using a FastDNA spin kit for soil (QIAGEN, Netherlands) according to the manufacturer’s instructions. The extracted DNA dissolved in 100 μL sterile double-distilled water and stored at −80°C until use. The V3–V4 region of the 16S rDNA was amplified with primers 341F (5′-CCTAYGGGRBGCASCAG-3′) and 806R (5′-GCCAATGGACTACHVGGGTWTCTAAT-3′). The sequencing library was constructed using Qiagen Gel Extraction Kit (Qiagen, Germany) and TruSeq^®^DNA PCR-Free Sample Preparation Kit (Illumina, United States), following manufacturers’ recommendations. The library was qualified on a Qubit^®^ 2.0 Fluorometer (Thermo Scientific, United States) and Agilent Bioanalyzer 2100 system (Agilent, United States). Finally, the library was sequenced using the Illumina HiSeq 2500 platform and 250 bp paired-end reads were generated.

The raw reads were analyzed using Mothur (Schloss et al., 2009) following the standard operating procedure. After assembly of the raw reads (Magoč and Salzberg, 2011), quality filtering (Bokulich et al., 2013), and chimeric sequence deletion (Edgar et al., 2011), an average of 33,380 effective reads were generated. Uparse (Uparse v7.0.1001)^1^ (Edgar, 2013) was used to cluster the effective tags and generate an operational taxonomic unit (OTU) with an identity threshold of 97%. The OTUs with only one sequence were removed, and the rest OTUs were annotated by the SILVA database (Quast et al., 2013). Relative abundance for each taxonomic level was calculated using R software (R Core Team, 2013), following OTU assignment and contaminative OTU deletion. All samples were rarefied to a sequencing depth of least-abundant sample for further analysis using QIIME (Caporaso et al., 2010). Rarefaction was performed using the *phyloseq* package in R software (R Core Team, 2013), and the diversity indices (Shannon, Simpson, Chao, and Ace) were estimated from the rarefied data using the *vegan* package in R software.

### Statistical Analysis

All experiments were conducted in triplicate except for crude oil bioremediation simulation tests. Data analysis and figures were conducted in Origin 9.0 and R software.

## Results and Discussion

### Bacteria Isolation and Identification

The isolated crude oil degraders were named as strains T-04 and 1-1. For strain T-04, colonies on LB medium appeared to be white, flat, and rough, with irregular edges. For strain 1-1, colonies on LB medium appeared to be white, convex, and smooth, with regular edges (Supplementary Figure S1).

**FIGURE 1:**
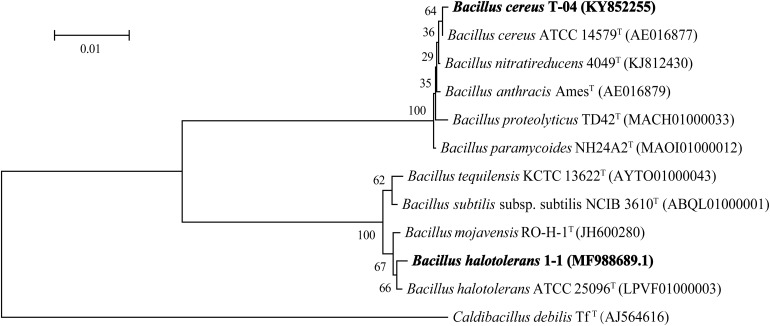
Neighbor-joining phylogenetic tree based on the 16S rRNA gene sequence analysis showing the relationships of strains T-04 and 1-1 within the genus *Bacillus*. Bootstrap percentages (based on 1000 replications) are indicated at the branch points. GenBank accession numbers are given in parentheses. *Endobacter medicaginis* M1MS02^T^ was used as the outgroup. Bar, 0.01 nucleotide substitutions per position.

The results of 16S rDNA sequence analysis suggested that strain T-04 possessed 99.66% sequence similarity with strain *Bacillus cereus* ATCC 14579 while strain 1-1 possessed 99.59% sequence similarity with strain *Bacillus halotolerans* ATCC 25096. The 16S rDNA sequences of both strains were submitted to the GenBank database under accession numbexr KY852255 for strain T-04 and MF988689.1 for strain 1-1. A phylogenetic tree based on the 16S rDNA sequences of each strain was constructed by neighbor-joining (Figure 1). Based on the morphology and phylogenetic analysis results, strain T-04 was suggested to be *Bacillus cereus* T-04 and strain 1-1 was suggested to be *Bacillus halotolerans* 1-1.

### Crude Oil Biodegradation Characteristics for Both Strains

**FIGURE 2:**
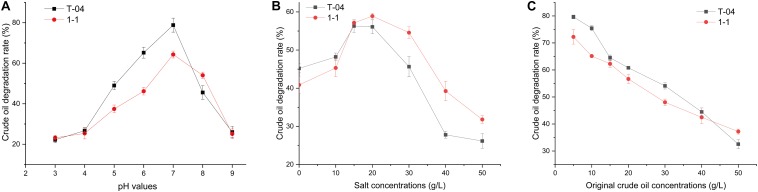
Crude oil degradation rates of each strain under different conditions. The crude oil degradation efficiencies of the isolates were tested: **(A)** under different original pH values (3.0, 4.0, 5.0, 6.0, 7.0, 8.0, and 9.0, with crude oil concentration of 5 g/L and salt concentration of 20.0 g/L); **(B)** under different original salt concentrations (0, 10, 15, 20, 30, 40, 50, and 60 g/L, with pH 7.0 and crude oil concentration of 5 g/L); **(C)** under different original crude oil concentrations (5, 10, 15, 20, 30, 40, and 50 g/L, with pH 7.0 and salt concentration of 20.0 g/L). All experiments were conducted in triplicate.

To understand the crude oil degradation characteristics for strains T-04 and 1-1, the degradation rate of crude oil for each strain was tested under different pH, salinity, and original crude oil concentrations. The results are shown in Figure 2.

Environmental pH is one of the main physicochemical parameters playing an important role in bacterial growth and metabolism. Strains T-04 and 1-1 showed different crude oil degradation performance under different pH conditions. The highest degradation rate appeared at neutral pH, and the degradation rate reduced as the pH values increased or decreased (Figure 2A). A similar phenomenon has also been found in some other bacteria. The highest crude oil degradation rate was also observed at pH 7 for strains *Bacillus* sp. IOS1-7 and *Pseudomonas* sp. BPS1-8. However, at pH 9, strains *Bacillus* sp. IOS1-7 and *Pseudomonas* sp. BPS1-8 still possessed relatively high crude oil degradation ability (Sathishkumar et al., 2008). At pH 5, 6, and 7, the crude oil degradation rates of strain T-04 were extremely significantly higher than those of strain 1-1 (*P* = 0.002, 0.001, and 0.008, respectively). Moreover, strain 1-1 showed a significantly higher crude oil degradation rate at pH 8 than strain T-04 (*P* = 0.031). When the pH value was higher than 8 or less than 5, the crude oil degradation rates of both strains reduced sharply. The sharply decreased crude oil degradation rate under acidic or alkaline conditions implied that the performance of strains could be mainly influenced by the soil pH.

The soil salinity has been observed to have an important influence on the crude oil biodegradation (Mille et al., 1991), so we tested the crude oil biodegradation of both strains under different salt concentrations. Both strains showed similar crude oil degradation rate at a salt concentration of 15 g/L (Figure 2B). Strain T-04 showed a higher crude oil degradation rate at low salinity than strain 1-1, while strain 1-1 had a stronger crude oil depletion capacity than strain T-04 at salt concentrations higher than 5 g/L. The differences in crude oil degradation rates for both strains at salt concentrations of 30, 40, and 50 g/L were significant (*P* = 0.023, 0.016, and 0.034, respectively). The salinity tolerance tests implied that strain 1-1 was more suitable for the biodegradation of crude oil contamination in high-salinity environments.

The higher the concentration of crude oil contamination, the more serious the inhibition of the microorganisms. The biodegradation results of different original crude oil concentrations also supported this point. As the original crude oil concentration was increased from 5 to 50 g/L, the degradation rates of crude oil dramatically decreased from 70–80% to 30–40% (Figure 2C). A similar result was also reported in some other crude oil degraders isolated from an oil-contaminated field (Sathishkumar et al., 2008). At original crude oil concentrations of 10 and 30 g/L, strain T-04 possessed significantly higher crude oil degradation rates than 1-1 (*P* = 0.034 and 0.025, respectively).

### Crude Oil Biodegradation Under Laboratory Conditions

**FIGURE 3:**
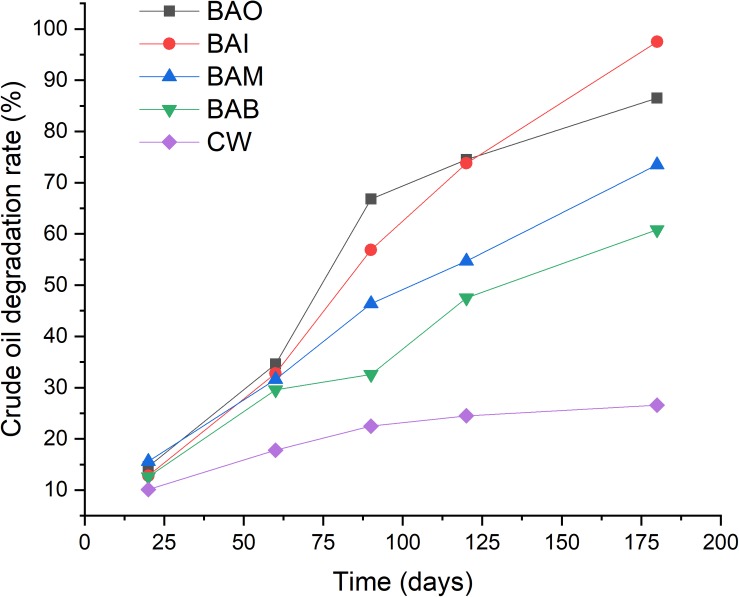
Bioremediation simulation tests of crude-oil-contaminated soil. CW, contaminated soil plus 200 mL sterilized water; BAI, bacterial inoculum with 200 mL MS medium; BAO, bacterial inoculum with 200 mL MO medium; BAM, bacterial inoculum with 200 mL MX medium; BAB, pure bacteria with 200 mL sterile water.

Simulation tests were conducted in order to evaluate the effect of the two strains in soils contaminated by crude oil, and the results showed that the addition of the two mixed strains could accelerate the crude oil depletion in the soil (Figure 3). The natural degradation of crude oil in soils was very slow, and only about 25% of the crude oil was degraded after 180 days. The results also showed that the natural degradation of crude oil in soils was almost stopped after 90 days. Soil microorganisms play a key role in the major biogeochemical nutrient cycles, including those of organic carbon, nitrogen and phosphorus (Stevenson and Cole, 1999). However, when the soil physicochemical properties were not suitable for bacteria growth, the bacteria could enter into a viable but non-culturable (VBNC) state (Su et al., 2018, 2019). The crude oil contamination could inhibit the microbial activity and slow the degradation progress by microorganisms.

Although the nutrients were different (i.e., BAO, BAI, and BAM), the addition of the mixed strains promoted the biodegradation of crude oil in soils. The addition of pure bacteria with sterile water also promoted the oil biodegradation (BAB). However, the extent of oil degradation promotion was the lowest for BAB among the four treatments. The lowest crude oil degradation of BAB might be explained by the bacteria inoculated into crude oil contaminated soil entered into VBNC state. A test of the GFP tagged variant of *Novosphingobium* sp. LH128 inoculated in soil showed about 99% reduction in Colony Forming Units (CFU) while the microscopic results showed that the GFP-expressing cells were identical to the expected initial cell density, which indicated that the inoculated bacteria entered into a VBNC-like state (Fida et al., 2017).

The addition of MI medium with the mixed bacteria showed the highest crude oil degradation rate of 97.5% after 180 days while the addition of MO medium led to a rate of 86.5%. The added nutrients might play roles like resuscitation-promoting factor and strengthen the bioprocess of crude oil degradation (Su et al., 2018) and different nutrients kinds could have different functions. Many reports have proven the enhancement of crude oil degradation with specific bacteria and nutrients addition (Rahman et al., 2002; Xia et al., 2017, 2019). The difference was only reflected in the crude oil degradation rate, which was mainly based on the degradation ability of the strains. The difference was only reflected on the crude oil degradation rate which mainly based on the degradation ability of the strains and the different kinds of nutrients.

### Shifts in Soil Microbial Community

To understand the microbial community of each group at the end of simulation tests, an Illumina HiSeq sequencing method was used to investigate the microbial diversity. A total of 329,809 raw reads were generated from the Illumina HiSeq sequencing. After quality control and chimera removal, a total of 3,057,015 clean reads remained for OTU generation. The Q20 values for the six samples ranged from 84.13 to 86.01, indicating high quality of the Illumina sequencing. The specific sequencing results for each sample are shown in Table 2. The sample SOIL represents the original clean soil without any treatment. The Illumina 16S rRNA gene sequence data have been deposited in the NCBI Sequence Read Archive under accession number SUB6674142.

**TABLE 2:** Overview of the sequencing data.

**Sample name**	**Raw reads**	**Clean reads**	**Base (nt)**	**AvgLen^#^ (nt)**	**Q20**	**GC%**	**Effective%**
SOIL	52,661	50,062	20,849,354	416	85.13	55.98	95.06
CW	51,744	50,162	20,649,659	411	86.01	56.89	96.94
BAI	63,642	59,201	24,797,577	418	85.57	54.31	93.02
BAO	55,193	50,157	21,063,094	419	84.53	52.77	90.88
BAB	55,486	50,034	20,975,563	419	83.41	53.82	90.17
BAM	51,083	47,399	20,004,570	422	84.13	53.81	92.79

*^#^AvgLen represents average length.*

**FIGURE 4:**
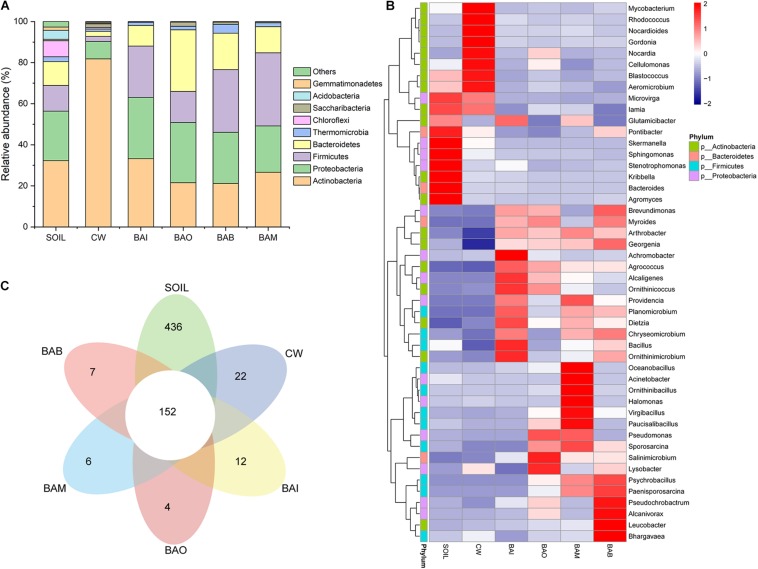
Microbial community changes in different treatment samples. SOIL represents original clean soil without crude oil contamination. **(A)** The relative abundance of each sample at phylum level. **(B)** Heatmap of genera with relative abundance greater than 0.01. **(C)** Flower map showing the core and specific operational taxonomic units (OTUs) in each sample.

**FIGURE 5:**
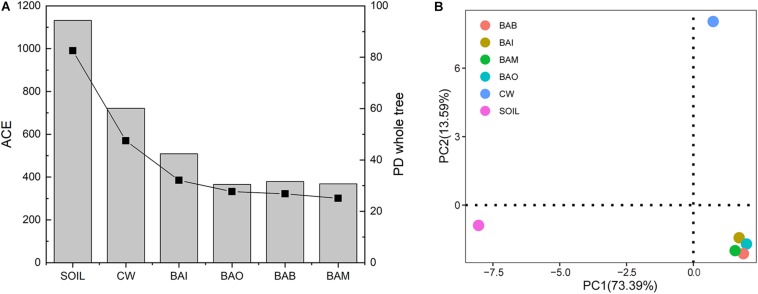
Alpha and beta diversity of samples in the bioremediation simulation tests. **(A)** Alpha diversity of abundance-based coverage estimator (ACE) and phylogenetic distance (PD) whole-tree index of each sample. The bar represents the ACE index and the square and line represents the PD whole-tree index. **(B)** Principal component analysis (PCA) of the six samples.

The microbial community composition at the phylum level possessed huge differences between SOIL and CW samples. The crude oil contamination in soil strongly influenced the proportion of Actinobacteria. The enrichment of Actinobacteria in the oil-contaminated soil was also reported in other research (Jung et al., 2016; Wu et al., 2017). After bioremediation (BAI, BAO, BAB, and BAM), the soil microbial community composition at phylum level was recovered to be similar to SOIL, in which Actinobacteria, Proteobacteria, Firmicutes, and Bacteroidetes were the dominant phyla (Figure 4A). However, the heatmap of the selected genera (relative abundance > 0.01) showed the differences among the SOIL, CW, and bioremediation samples (Figure 4B). As expected, the relative abundance of some genera which may play important roles in crude oil biodegradation increased in the bioaugmentation treatment groups, such as *Arthrobacter*, *Pseudomonas*, *Bacillus*, *Alcaligenes*, *Acinetobacter*, and so on (Ollivier and Magot, 2005; Banerjee and Ghoshal, 2010; Pandey et al., 2016; Tao et al., 2017). There were only 152 core OTUs in all samples, while the SOIL sample possessed the largest number of specific OTUs at 436, which is far more than the other five samples (CW at 22, BAI at 12, BAO at 4, BAM at 6, BAB at 7) (Figure 4C). The fact that the largest specific OTUs were in SOIL implies the great microbial diversity in the original clean soil. The crude oil contamination could play important roles in the microbial composition and dramatically decrease the soil microbial diversity. The abundance-based coverage estimator (ACE) of the SOIL sample was the highest, and a dramatic decrease was seen in the crude-oil-contaminated soil. Not only could the bioremediation treatment not recover the ACE index of the contaminated soil, it was even seen to lower it (Figure 5A). Because ACE index represents the species abundance, the SOIL sample showed the highest species abundance among the six samples. A similar trend appeared for the phylogenetic distance (PD) whole-tree index (Figure 5A), which represents the species diversity of the sample. The ACE index and PD whole-tree index revealed that the original clean soil possessed the highest species abundance and diversity. However, these experimental results were contrary to expectation and the ACE and PD whole tree index of bioaugmentation treatment groups were lower than the CW group. The addition of bacteria degraders and nutrients might contribute to the decrease of species abundance and diversity. Principal component analysis (PCA) also showed the huge difference among SOIL, CW, and bioremediation treatment samples (Figure 5B). The bioremediation treatment samples showed little difference in the microbial community composition. Although the bioremediation treatment can accelerate the degradation rate of crude oil, it cannot recover the soil microbial community in short term. Thus in the bioremediation of crude oil environmental, it will take a long time for microbial community recovery and more efforts should be put into this field.

## Conclusion

Two crude-oil-degrading strains, T-04 and 1-1, were isolated from crude-oil-contaminated soils. Morphology and phylogenetic analysis suggested they belonged to genus *Bacillus*, and they were designated as *Bacillus cereus* T-04 and *Bacillus halotolerans* 1-1. Both strains possessed high efficiencies for crude oil depletion (about 60–80% crude oil was removed in 20 days). The highest crude oil depletion in simulation tests reached nearly 100%.

Crude oil contamination can dramatically decrease the soil bacteria abundance and diversity. The bioremediation treatment could not recover the soil microbial ecology in the short term. However, the bioremediation could recover the proportion of the dominant phyla to a certain extent. Thus, the results showed that the enhancement of crude oil depletion in bioremediation, but the recovery of soil microbial ecology would take a long time.

## Data Availability Statement

The 16S rRNA Illumina libraries were deposited in the NCBI small read archive (SRA) data set under the BioPoject accession number of PRJNA594790 and the SRA accession numbers SRR10687677-SRR10687682.

## Author Contributions

ZD and XL designed the study. KC and FG conducted the research. YJ and XL analyzed the data. XL wrote the manuscript.

## Conflict of Interest

The authors declare that the research was conducted in the absence of any commercial or financial relationships that could be construed as a potential conflict of interest.

## Footnotes

1 http://drive5.com/uparse/
