# Supplementary material for: Petroleum Depletion Property and Microbial Community Shift After Bioremediation Using Bacillus halotolerans T-04 and Bacillus cereus 1-1
Source: Front Microbiol. 2020 Mar 5;11:353. doi: 10.3389/fmicb.2020.00353 (PMC7066087; doi:10.3389/fmicb.2020.00353)
Supplement: Supplementary file 1 [file Data_Sheet_1.PDF]

Supplementary Material

1 Supplementary Figures and Tables

1.1 Supplementary Figures

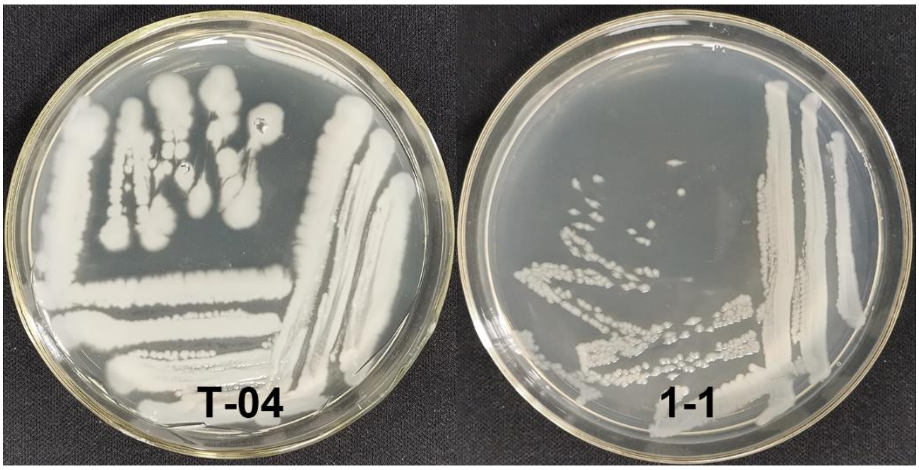

Supplementary Figure 1. Morphology of strains *Bacillus cereus* T-04 and *Bacillus halotolerans* 1-1 grown on LB medium.
